# In search of a general theory of species’ range evolution

**DOI:** 10.1371/journal.pbio.2006735

**Published:** 2018-06-13

**Authors:** Tim Connallon, Carla M. Sgrò

**Affiliations:** School of Biological Sciences, Monash University, Melbourne, Australia

## Abstract

Despite the pervasiveness of the world’s biodiversity, no single species has a truly global distribution. In fact, most species have very restricted distributions. What limits species from expanding beyond their current geographic ranges? This has been classically treated by ecologists as an ecological problem and by evolutionary biologists as an evolutionary problem. Such a dichotomy is false—the problem of species’ ranges sits firmly within the realm of evolutionary ecology. In support of this view, Polechová presents new theory that explains species’ range limits with reference to two key factors central to both ecological and evolutionary theory—migration and population size. This new model sets the scene for empirical tests of range limit theory and builds the case for assisted gene flow as a key management tool for threatened species.

## Introduction

All species are restricted in their distributions. Traditionally, attempts to understand why this is the case have taken either an ecological or evolutionary perspective. Yet species’ range evolution is the quintessential evolutionary ecology problem—one that cannot be understood in purely ecological or evolutionary terms. Population size dynamics, including the distribution of individuals across a species’ range, fall within the classical domain of ecology, which is concerned with interactions between organisms and environment across contemporary timescales. On the other hand, the determinants of population size dynamics (i.e., birth and death rates) are evolvable features of populations, reflecting adaptation (or maladaptation) to environmental conditions within the range.

Nevertheless, the act of constructing a coherent theory of species’ range evolution was never destined to be easy. The first obstacle is the divide between evolutionary genetics and population ecology, which follow from disparate traditions. As Roughgarden [1] puts it:

The fusion of population genetics with population ecology can be compared to a prearranged marriage between partners who speak different languages. Although both families agree that the marriage is advantageous, it is somewhat difficult to achieve because of cultural difference between geneticists and ecologists.

The second obstacle is one of complexity. A minimally complete theory for species’ range evolution must unite population dynamics with features of environmental variation, evolution, and genetics that are challenging to model in combination. The ingredients of such a theory must accommodate four biological realities:

1. Species’ ranges are distributed across two dimensions of geographic space. Locations along the two major axes of a species’ range (latitude and longitude) vary in their environmental conditions and local population densities.
2. Dispersal and local adaptation affect population dynamics. Local population densities are affected by rates of immigration and emigration and by the degree to which their inhabitants are adapted to conditions of the local environment.
3. The genetic basis of traits is polygenic and evolvable. Many genes contribute to the traits that underpin local adaptation and species’ ranges. Evolution acting on these genes leads to changes in both the means and genetic variances of traits affecting adaptation.
4. Nature is somewhat unpredictable. Genetic drift—random evolutionary changes of traits and gene frequencies—reduces genetic variation and evolutionary potential in parts of the range where population size is consistently small.

Although these ingredients were identified long ago as potentially important for species’ range evolution [2,3], mathematically formalising them into a single, coherent model, and then extracting straightforward predictions from that model, is extremely challenging. Indeed, some of the model ingredients listed above are difficult to work with in isolation and unsurprisingly represent later arrivals in the origins of classical population genetics (e.g., [4–7]).

Despite these obstacles, a comprehensive theory of range limit evolution is desirable, indeed essential, for three reasons. Such a theory (i) identifies the most important processes affecting range limits, (ii) guides empirical research into the causes of range limits in nature, and (iii) supports efforts of conservation biologists to manage threatened populations, which face similar constraints to persistence as do marginal populations within a species’ range.

In the following primer, we review ecological and evolutionary factors that affect species’ range dynamics and efforts to formalize these factors into a general mathematical theory of species’ range evolution. We close by discussing an exciting theoretical study by Polechová [8] (this issue of *PLoS Biology*), which brings together all four of the above ingredients into a powerful new model for the evolution of species’ range limits.

## Ecological and evolutionary drivers of species’ range limits

Species’ range distributions vary widely among species. Some span continents, whereas others are endemic. Some are continuously distributed, while others are fragmented. Species’ ranges also differ in the sharpness of their edges. Some species have high population densities at the center of the range and gradually decline towards their margins [9,10]. Others show consistently high density across the range before they abruptly end [10,11]. The goal of research on range limits is to explain why species vary in the broad features of their geographic distributions and to understand the evolutionary and ecological mechanisms leading to population failure at and beyond current range margins.

In some cases, range limits reflect historical contingencies rather than mechanistic connections between the species’ range and ecological niche. For example, habitats beyond current range margins may be suitable for a species, yet limited dispersal [12] or density-dependent constraints to establishment (i.e., Allee effects [2]) hinder establishment of the species beyond its current range. In such cases, transplant experiments are expected to show positive population growth in locations beyond current range boundaries. And indeed, such findings are relatively common (i.e., approximately 25% of experiments reviewed in [12]).

In other cases, range distributions genuinely reflect the ecological niche of the species. As such, populations of the species cannot persist in locations beyond range margins because death rates outstrip birth rates in these regions [12]. The causes of population failure beyond range margins could reflect hard limits of the species’ physiological tolerance of environmental extremes [13]. But species’ range limits—and their proximate causes—need not be fixed. Evolution can, in principle, permit local populations of a species to improve in competitive ability or physiological tolerance and thereby expand the species’ range beyond its current limits. In this case, the question is why marginal populations fail to locally adapt and thereby expand the species’ range.

## The evolution of species’ range limits in one dimension

Our understanding of the evolutionary drivers of species’ range limits has expanded greatly over the last two decades, owing to sustained efforts to merge population dynamics theory with population genetic scenarios of adaptation in spatially varying environments. We now recognize three key factors that determine whether a species’ distribution will expand to occupy an expansive range or collapse to a restrictive one. These factors are important in species that are distributed across a one-dimensional range (e.g., a river or narrow valley running north to south)—a simplifying assumption in virtually all previous models of species’ range evolution.

### The cost of migration

Environmental conditions change continuously across space; species face the challenge of locally adapting to these spatially variable conditions. In this context, migration can be costly, as it constrains local adaptation. Kirkpatrick and Barton [3] were the first to show that environmental change and high rates of dispersal from the range centre towards the range margin—which combine to determine the cost of migration—can exert limits on species’ range breadth. When the cost of migration is high compared to the amount of genetic variation in traits affecting local adaptation and population growth, then species’ ranges are limited (extinction is also possible). Gradual environmental change and low costs of migration allow for local adaptation everywhere and expansion of the species’ range.

### Evolution of genetic variation

Early theory assumed that genetic variation was constant across the range, and in such cases, the cost of migration is the primary determinant of range breadth [3,14]. The story changes substantially when genetic variation evolves across the range. In the absence of drift, migration inflates genetic variance and the evolutionary potential of marginal populations, leading to perpetual expansion of the range [15]. With declining population densities—either globally or locally within the species’ range—genetic drift can substantially reduce standing genetic variation, leading to the emergence of range limits or population fragmentation [16].

### Effects of ‘genetic architecture’

The effect of genetic drift on range limits hinges upon the genetic basis of phenotypes under selection—whether genetic variants have large or small phenotypic effects on trait expression [16,17]. A species’ range will expand when the ‘efficacy of selection’ at the range edge—a function of the local population size, migration rate, strength of selection on the trait, and effect size of genetic variants on trait expression—is large relative to the rate of environmental change across space. In cases for which local population sizes are small and genetic drift is strong, range expansions are unlikely when the phenotypic effects of mutations are small, whereas large-effect mutations make range expansions more permissible [16] (also see [17]).

## Towards a general theory of species’ range limits

Current theories of range limit evolution overwhelmingly focus on one-dimensional ranges. Yet, given that most species do not occupy one-dimensional habitats, the question is whether the ‘rules’ of species’ range evolution that apply to one-dimensional ranges will also apply in two-dimensional ranges. Moreover, does the added complexity of such a model lead to hopelessly more-complicated dynamics and completely preclude analytical progress with the model? On the contrary, Polechová [8] demonstrates that results for the full model (including all four ingredients listed above) are actually simpler than those arising in one-dimensional habitats.

Polechová [8] first developed analytical guidelines by analysing a set of reaction-diffusion equations that describe dynamical changes in population size and the frequencies of alleles of a trait responding to local selection across the two-dimensional range. Migration is random, and the environment (and corresponding trait optimum) changes monotonically along one dimension of the range. The behaviour of the model is captured by four compound parameters. The first corresponds to ‘the cost of migration’ from earlier species’ range models [16] (see above). The second, the ‘neighbourhood size’, depicts the role of genetic drift in reducing genetic variation (neighbourhood size is inversely proportional to the intensity of drift). The remaining parameters reflect the genetic basis of the trait and are functions of the mutation rate and the phenotypic effect sizes (respectively) per locus affecting trait expression.

**Fig 1:**
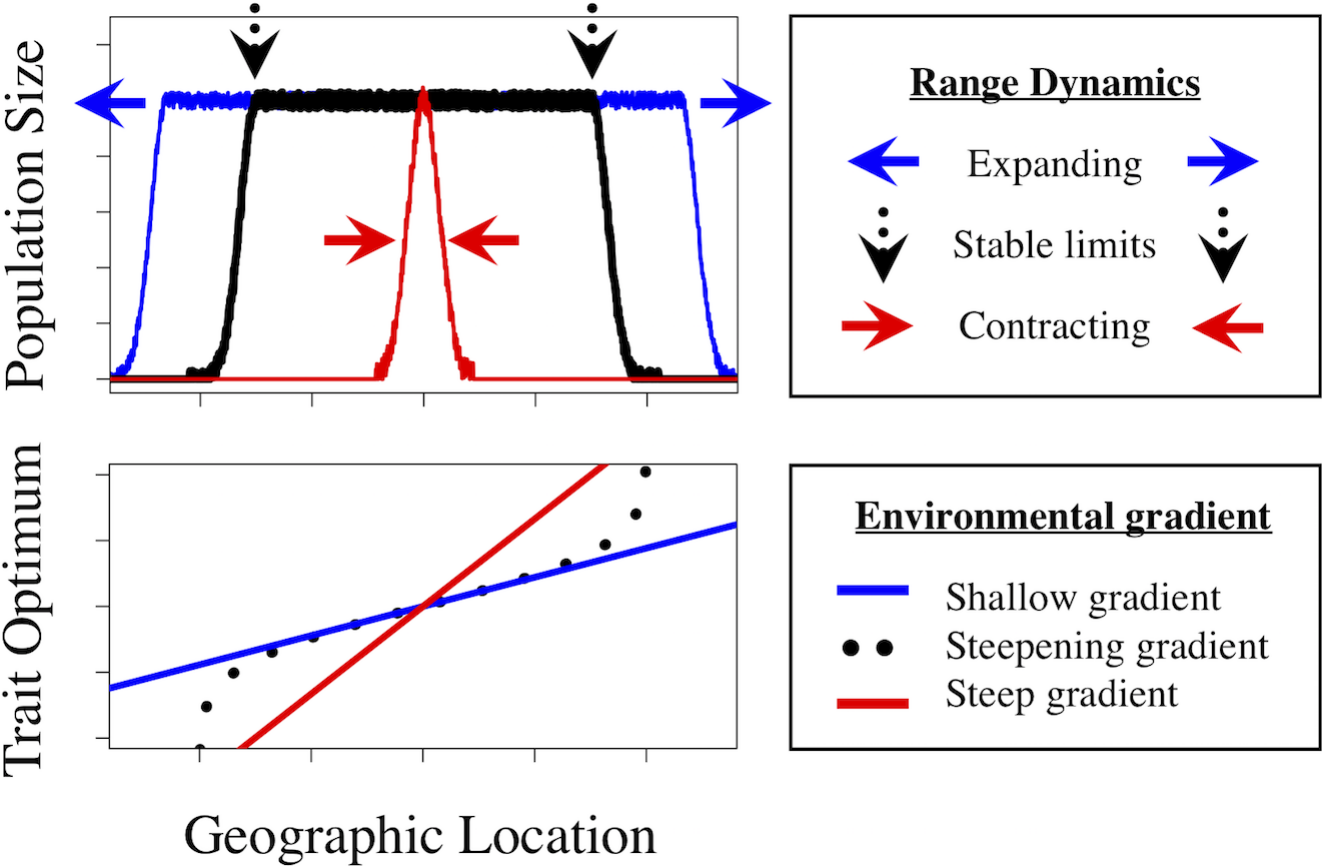
Species’ range dynamics depend on the balance between environmental change and genetic drift. Specifically, range limit evolution depends on two compound parameters: (1) the ‘cost of migration’, which is proportional to the mean dispersal distance of individuals of the species, as well as the steepness of the environmental gradient (i.e., the rate at which a trait optimum changes across space), and (2) ‘neighbourhood size’, which is inversely proportional to the local intensity of genetic drift. As Polechová [8] demonstrates, species’ range dynamics, including the location and stability of range limits, are predicted by the relative magnitude of neighbourhood size versus the steepness of the environmental gradient. For example, with a shallow environmental gradient relative to neighbourhood size, the species’ range expands to fill all of the available habitat (blue plots). With a steep environmental gradient relative to neighbourhood size, the species’ range contracts, leading to extinction or a restricted or fragmented range (red plots). With a steepening environmental gradient (black plots), the neighbourhood size can be sufficiently large to maintain local adaptation and high population density through most of the range (see the regions between the vertical arrows). However, as the environmental gradient steepens, the threshold neighbourhood size required to maintain local adaptation increases. Eventually, the neighbourhood size falls below the threshold for local adaptation (black arrows note the threshold), and abrupt range limits arise.

As it turns out, the first two parameters are the only ones that matter [8]. When the rate of environmental change is constant across space, the species’ range collapses or becomes fragmented when neighbourhood size is small relative to the environmental gradient (Fig 1, red), or the range expands indefinitely when the neighbourhood size is high, and drift becomes negligible (Fig 1, blue). Other forms of environmental change, including a steepening environmental gradient with distance from the range centre, can lead to continuous ranges with high population density throughout the range and abrupt range limits in marginal populations in which neighbourhood size dips below the threshold for range expansion (Fig 1, black). Intriguingly, these results are roughly independent of the details of the genetic basis of the trait itself—and in that sense, generate much simpler predictions than prior models of one-dimensional species’ ranges.

The study provides the most biologically complete theoretical analysis of species’ range evolution to date, with two major messages. First, empirical tests of evolutionary limits to species’ range breadth are more hopeful than implied by prior theory. By relaxing the need to account for details of genetic architecture—which are difficult enough to account for in laboratory contexts—researchers can invest money and time into evaluating the role of drift (through patterns of neutral diversity) and costs of migration (through transplant experiments) as drivers of range limits. Second, from a species conservation perspective, the study provides a strong justification for management strategies that promote gene flow into marginal and threatened populations. As Polechová [8] demonstrates, for marginal populations, the beneficial effects of gene flow (elevated genetic diversity) exceed the costs (migration load), resulting in a net benefit of facilitating dispersal. Conservation biologists have been slow to embrace assisted gene flow as a management option. Yet Polechová’s work [8], combined with the increasing evidence of the success of assisted gene flow in threatened species management [18], should act to galvanise action on this front.

## References

[1] Roughgarden J. *Theory of population genetics and evolutionary ecology*: *An introduction* New York: Macmillan Publishing Co.; 1979.

[2] Haldane JBS. The relation between density regulation and natural selection. Proc Roy Soc Lond B 1956;145: 306–308. 13359386 10.1098/rspb.1956.0039

[3] Kirkpatrick M, Barton NH. Evolution of a species’ range. Am Nat. 1997;150: 1–23. doi: 10.1086/286054 18811273 10.1086/286054

[4] Haldane JBS. The theory of a cline. J Genet. 1948;48: 276–284. 10.1007/BF02986626 18905075

[5] Kimura M. A stochastic model concerning the maintenance of genetic variability in quantitative characters. Proc Natl Acad Sci USA 1965;54: 731–736. 5217452 10.1073/pnas.54.3.731 PMC219735

[6] Nagylaki T. Random genetic drift in a cline. Proc Natl Acad Sci USA 1978;75: 423–426. 16592484 10.1073/pnas.75.1.423 PMC411261

[7] Barton NH, Depaulis F, Etheridge AM. Neutral evolution in spatially continuous populations. Theor Pop Biol. 2002;61: 31–48. 11895381 10.1006/tpbi.2001.1557

[8] Polechová J. Is the sky the limit? On the expansion threshold of a species’ range. PLoS Biol. 2018;16(6): e2005372 doi: 10.1371/journal.pbio.2005372 29906294 10.1371/journal.pbio.2005372 PMC6021114

[9] Brown JH. On the relationship between abundance and distribution of a species. Am Nat. 1984;124: 255–279.

[10] Sagarin RD, Gaines SD. The ‘abundant centre’ distribution: to what extent is it a biogeographical rule? Ecology Letters. 2002;5: 137–147.

[11] Griggs RF. Observations on the behaviour of some species at the edges of their ranges. Bulletin of the Torrey Botanical Club 1914;41: 25–49.

[12] Hargreaves AL, Samis KE, Eckert CG. Are species’ range limits simply niche limits writ large? A review of transplant experiments beyond the range. Am Nat. 2014;183: 157–173. doi: 10.1086/674525 24464192 10.1086/674525

[13] Sexton JP, McIntyre PJ, Angert AL, Rice KJ. Evolution and ecology of species range limits. Annu Rev Ecol Evol Syst. 2009;40: 415–436.

[14] Case TJ, Taper ML. Interspecific competition, environmental gradients, gene flow, and the coevolution of species’ borders. Am Nat. 2000;155: 583–605. doi: 10.1086/303351 10777432 10.1086/303351

[15] Barton NH. Adaptation at the edge of a species’ range In: Silvertown J, Andonovics J, editors. Integrating Ecology and Evolution in a Spatial Context. Blackwell Science; 2001 pp. 365–392.

[16] Polechová J, Barton NH. Limits to adaptation along environmental gradients. Proc Natl Acad Sci USA. 2015;112: 6401–6406. doi: 10.1073/pnas.1421515112 25941385 10.1073/pnas.1421515112 PMC4443383

[17] Gilbert KJ, Whitlock MC. The genetics of adaptation to discrete heterogeneous environments: frequent mutation or large-effect alleles can allow range expansion. J Evol Biol. 2017;30: 591–602. doi: 10.1111/jeb.13029 27992089 10.1111/jeb.13029

[18] Weeks AR, Heinze D, Perrin L, Stoklosa J, Hoffmann AA, van Rooyen A, Kelly T, Mansergh I. Genetic rescue increases fitness and aids rapid recovery of an endangered marsupial population. Nat Comm. 2017;8: 1071. 10.1038/s41467-017-01182-3 PMC5715156 29057865

